# First Detection of *Encarsia smithi* in Italy and Co-Occurrence with *Eretmocerus iulii*: A Case of Unintentional Introductions and New Associations with the Invasive Species *Aleurocanthus spiniferus*

**DOI:** 10.3390/insects16090891

**Published:** 2025-08-27

**Authors:** Gianluca Melone, Lucia Andretta, Feliciana Pica, Francesco Pio Donnarumma, Roberta Ascolese, Francesco Nugnes, Stefania Laudonia

**Affiliations:** 1 Institute for Sustainable Plant Protection (IPSP)—CNR, P.le Enrico Fermi 1, 80055 Portici, Italy; gianluca.melone1998@gmail.com (G.M.); felicianapica@cnr.it (F.P.); robertaascolese@cnr.it (R.A.); 2 Department of Agricultural Sciences, University of Naples Federico II, 80055 Portici, Italy; lucia.andretta@unina.it (L.A.); francescop.donnarumma@gmail.com (F.P.D.); laudonia@unina.it (S.L.); 3 Department of Biology, University of Naples Federico II, 80126 Napoli, Italy; 4 Center for Studies on Bioinspired Agro-Environmental Technology, BAT Center, University of Naples Federico II, 80055 Portici, Italy

**Keywords:** biological control, parasitoid wasps, citrus agroecosystems, Orange Spiny Whitefly

## Abstract

The occurrence of the invasive Orange Spiny Whitefly (*Aleurocanthus spiniferus*) has expanded rapidly in Italy, prompting responses from both native and unintentionally introduced natural enemies. Through field monitoring, morpho-molecular identification, and phenological analysis, a multi-species parasitoid complex acting on this pest was revealed. In addition to the predominant activity of *Eretmocerus iulii*, this study reports the first European detection of *Encarsia smithi*, genetically confirmed as belonging to haplogroup I, a lineage previously associated with *A. spiniferus* in Asia. Occasional parasitization by *Cales noacki* was also observed. Seasonal patterns suggest a possible ecological complementarity between *Er. iulii* and *E. smithi*, which may contribute to the suppression of *A. spiniferus* populations. Although climatic variables showed weak correlations with infestation or parasitism rates, a slight positive relationship was observed between rainfall and whitefly abundance. The increasing biocenotic complexity, reflected by the diversity and interactions among parasitoid species, indicates an emerging ecological balance. These findings underscore the potential role of unintentional biological control in supporting agroecosystem resilience.

## Simple Summary

The Orange Spiny Whitefly is an invasive insect that feeds on plant sap and is increasingly spreading across Italy, damaging citrus and ornamental plants. This study reports the first discovery in Europe of a minute parasitoid, *Encarsia smithi*, which was found attacking this pest. The wasp appeared alongside another recently identified species, *Eretmocerus iulii*. Both parasitoids were found to naturally limit whitefly populations without any human intervention. The presence of *E. smithi* in Italy may be the result of an unintentional introduction, possibly through plant trade. These findings highlight the role of naturally occurring beneficial insects in helping control invasive pests and reducing the need for chemical treatments. Understanding how these species interact with pests can support more sustainable and environmentally friendly approaches to plant protection.

## 1. Introduction

The genus *Aleurocanthus* Quaintance and Baker (Hemiptera: Aleyrodidae) includes almost 100 species worldwide, some of which are pests of cultivated plants [1,2,3]. Among these, in Italy, there are 2 species of the genus: the Tea Spiny Whitefly (TSW), *Aleurocanthus camelliae* Kanmiya and Kasai and the Orange Spiny Whitefly (OSW), *Aleurocanthus spiniferus* (Quaintance) [4,5]. The first is probably native to Taiwan or China and has spread to Japan, Indonesia, the Netherlands, and Italy [4,5]. OSW is native to Southeast Asia and has spread to most tropical and subtropical regions, including Africa, Australia and the Pacific Islands. After the first report in Italy of OSW [6], confined to the Province of Lecce, the whitefly has been found in several other Italian areas [7,8] and in other Mediterranean countries [7,9,10], being currently listed as a quarantine pest for the European and Mediterranean area [3]. Molecular characterization of some Italian populations, together with an Albanian population, revealed two haplotypes currently invading Europe, both belonging to a haplogroup previously identified in China [7,11].

During early surveys accompanying the species’ spread across southern Italy, predation on OSW nymphs by the autochthonous *Clitostetus arcuatus* (Rossi) (Coleoptera: Coccinellidae: Coccidulinae) was documented [12], providing the first evidence of natural enemy activity against this invasive whitefly. Among exotic ladybird predators, two species of the tribe Serangiini, closely associated with whiteflies, have been recorded in Italy. The first exotic predator documented was *Delphastus catalinae* Horn [7], followed by *Serangium montazerii* Fürsch, which was recently reported [13]. Although both species are generally polyphagous, their activities have notably concentrated on the family Aleyrodidae. An important advance in understanding the natural enemies complex of *A. spiniferus* was the recent detection in Italy and subsequent taxonomic description of the parasitoid wasp *Eretmocerus iulii* Laudonia et Melone (Hymenoptera: Chalcidoidea: Aphelinidae) [8,14]. However, during field observations to assess the impact of *Er. iulii* on *A. spiniferus* populations, additional parasitoid species were unexpectedly detected. These included, for the first time in Europe, *Encarsia smithi* (Silvestri) (Hymenoptera: Chalcidoidea: Aphelinidae) and a limited but confirmed parasitization activity by *Cales noacki* Howard (Hymenoptera: Chalcidoidea: Calesidae).

*Encarsia smithi* is a parasitoid species primarily associated with whiteflies of the genus *Aleurocanthus*. Originally described from China, it is considered to be of Oriental origin [15]. Its current distribution includes several Nearctic regions (both continental and insular), South Africa, and Japan, where the species has acclimatized following its use in classical biological control programs targeting *Aleurocanthus woglumi* Ashby [15]. Precisely due to this association, more recently, *E. smithi* has been included in feasibility assessments for pre-emptive classical biological control programs targeting *A. woglumi* in Europe. However, there is no documented evidence of intentional releases within the EU territory [16,17,18]. Considering these observations, the present study aims to investigate the current composition and seasonal dynamics of the parasitoid community associated with *A. spiniferus* in Italy. Particular emphasis is placed on the role of both native and unintentionally introduced natural enemies in the spontaneous regulation of OSW populations. To address this, a multidisciplinary approach was adopted, integrating field monitoring, phenological observations, and integrated morphological and molecular identification. Furthermore, molecular characterization of *E. smithi* was performed to determine its haplogroup affiliation and explore hypotheses regarding its geographic origin. This comprehensive strategy was designed to clarify the ecological significance of these interactions and assess their implications for sustainable biological control strategies against invasive whiteflies.

## 2. Materials and Methods

### 2.1. Field Collections

From January 2024 to July 2025, as part of the Phytosanitary Surveillance Project of the Campania Region (Regional Phytosanitary Coordination Unit—U.R.Co.Fi: Strengthening of the supervision activities and control of pests), a total of 895 technical inspections were conducted at 175 sites in 2024, and 550 inspections at 137 sites in 2025. These inspections focused on *Citrus* species, specifically *Citrus limon* (L.) Burm. f., *Citrus reticulata* Blanco, *Citrus sinensis* (L.) Osbeck, *Citrus × aurantium* (L.), and *Citrus clementina* Hort. ex Tanaka cultivated in specialized orchards, private gardens, and public parks. The primary objectives were to monitor the spread of *A. spiniferus* and to identify the presence of its natural enemies. In parallel, from March 2024 to January 2025, weekly sampling was conducted in urban orchards located in the municipality of Portici (Province of Naples, Campania Region—Table 1), covering approximately 24,700 square meters and 200 citrus plants. This activity aimed to monitor the behavior and phenology of *Er. iulii*. The orchards are situated within Gussone Park, part of the Department of Agricultural Sciences at the University of Naples Federico II, and were selected due to their accessibility and the total absence of phytosanitary treatments in the whole park. In addition, a single collection was conducted in Grottammare (province of Ascoli Piceno, Marche region) to investigate the presence of parasitization and identify any parasitoid species occurring at that site (Table 1).

In Portici, an average of 24 fully developed leaves were sampled per week. Between early March and late May, sampling was restricted to mature leaves on shoots developed during the previous autumn, which retained overwintering OSW populations. From late May onward, the sampling strategy included both these autumnal shoots and newly expanded spring shoots, depending on the phenology of the plant. This shift was intended to monitor the development of the pest on the foliage throughout the growing season of the year. Third (N3) and fourth (N4) instars were present during late autumn and winter, while adults (A) and all developmental stages were detected during the remaining months. Due to the coexistence of multiple parasitoid species, exit holes were not included in the dataset, as emergence could not be reliably attributed to a specific taxon. The same collection protocol was used for the occasional sampling event. All plant material was sealed in double plastic bags and transported to the Department of Agricultural Sciences (University of Naples Federico II, Portici), where it was stored in a ventilated container for further analysis.

### 2.2. Parasitoids Morpho-Molecular Characterization

Specimens collected for taxonomic studies were mounted on slides using a balsam-phenol medium [19]. Dichotomous keys from the literature were used for the identification of parasitoids [14,20,21,22,23,24]. They were also compared with the specimens deposited at the Filippo Silvestri Museum of the Department of Agricultural Sciences, University of Naples Federico II, Portici, Italy. Samples preliminarily ascribed to *Encarsia* spp. were subjected to genomic DNA extraction at the Institute for Sustainable Plant Protection (IPSP)-CNR (Portici, Italy), with a conservative Chelex-proteinase K protocol [14]. The obtained DNA was stored at −20 °C until use. The concentration and purity of extracted total DNA were checked with a UV-visible spectrophotometer (Nanodrop 2000—Thermo Fisher Scientific, Wilmington, DE, USA). Following DNA extraction, each sample was rinsed with distilled water and mounted on slides for morphological examination, performed according to previously described preparation methods.

Previous studies [11] highlighted two distinct phylogenetic groups within *E. smithi*: haplogroup I, primarily associated with OSW, and haplogroup II, predominantly linked to TSW. These groups, both found in Japan, show marked genetic differences, suggesting separate introduction and colonization events. Based on this evidence, molecular analyses targeting the same mitochondrial gene region have been performed to determine the haplogroup of the *E. smithi* specimens collected during this study. Hence, the mitochondrial cytochrome c oxidase subunit I (COI) (for an amplicon size of ~ 800 bp) was sequenced using primer pair C1-J-2195 and LN-2-3014 [25] with the thermocycling conditions as in [26]. PCRs were performed on a ProFlex™ PCR System thermocycler (Thermo Fisher Scientific, Wilmington, DE, USA). PCR products were checked on a 1.2% agarose gel and directly sequenced (BMR Genomics, Padova, Italy). Assembly, editing, and alignment of sequences were conducted using BioEdit 7.0 software [27]. To detect frameshift mutations and nonsense codons, sequences were virtually translated to amino acids using EMBOSS Transeq (www.ebi.ac.uk/Tools/st/emboss_transeq/ accessed on 30 April 2025). Edited and trimmed sequences were blasted against the homologous sequences available in GenBank and matched against the BOLD database (www.boldsystems.org, last access 15 January 2025). The generated sequences were deposited in GenBank databases, with accession numbers reported in Table 2.

### 2.3. Active Parasitization Rate

From March 2024 to January 2025, observations focused on *A. spiniferus* individuals from the second (N2) to the fourth (N4) instar. Particular attention was given to N2–N3 to assess the potential activity of idiobiont parasitoids. N4 were detached and either dissected to detect parasitoid immature stages or isolated in natural gelatin capsules (13.59 mm × 5.57 mm) to monitor adult emergence. Out of 1774 apparently healthy N4 collected during the study period, a subset was randomly selected for isolation. Selection was carried out by arbitrarily picking individuals from multiple infested leaves per sampling date, aiming to proportionally represent different colony sizes. On average, 41.03% of N4 were isolated, with a range of 13.33% to 86.67%, depending on whitefly abundance on the sampled leaves.

The isolated samples were stored at 25 ± 2 °C, 65 ± 10% relative humidity, and 16:8 (L:D) photoperiod until the emergence of the parasitoids. Soon after the emergence, the parasitoid adults were killed in 70% ethanol (Carlo Erba Reagents srl, Milan, Italy) and stored at room temperature for morphological observations. Some specimens were killed in absolute ethanol and stored at −20 °C for molecular analysis. Additionally, apparently healthy N4 were examined under a stereomicroscope (Leica MZ16, Wetzlar, Germany) to detect the presence of eggs and first instar larvae of *Er. iulii* on the ventral side of the host. Observations were also conducted by dissecting N4 on a slide in distilled water and glycerin (Carlo Erba Reagents srl, Milan, Italy). This procedure aimed to detect immature stages of parasitoids, including ectophagous forms (eggs and larvae externally attached to the host) and endophagous larvae developing inside the host body. Dissections were performed immediately after collection to assess ongoing parasitization activity, particularly by early-stage ectoparasitoids such as *Er. iulii*, which may be difficult to detect through emergence data alone, especially in cases of incomplete development or parasitoid mortality. The Active Parasitization Rate (APR%) was calculated using the formula:

$$\frac{n{}^{\circ}\;apparently\;parasitized\;host}{\left(n{}^{\circ}\;apparently\;parasitized\;hosts\;+\;n{}^{\circ}\;apparently\;healthy\;hosts\right)}\;\times \;100$$

Apparently parasitized hosts consisted exclusively of N4 stages of OSW, identified by visible evidence of parasitic activity, such as parasitoid developmental stages (e.g., eggs, larvae, pupae, or emerging adults). In contrast, emergence holes were not evaluated due to the inability to correlate them with the active host population at any given time.

### 2.4. Phenology

Data on the phenology of *Er. iulii* were collected from the site in Portici, where sampling has been performed on a weekly basis. To this end, for each collection, all N4 whiteflies were counted on each sampled leaf. The apparently parasitized N4 were counted due to the presence of eggs and newborn larvae of the ectophagous stage of the parasitoid. Meanwhile, the apparently healthy forms were partly isolated and partly dissected to evaluate the endophagous stages of the parasitoid. From the isolated N4, as described in the previous paragraph, the number of emerged adults of the parasitoids was recorded.

### 2.5. Climate Data and Statistical Analyses

Temperature and precipitation trends in the Portici area were analyzed to evaluate their influence on the phenology of *A. spiniferus* and the APR%. Meteorological data were obtained from the online database of the Civil Protection Organization of the Campania Region (https://centrofunzionale.regione.campania.it) (accessed on 3 April 2025) and referred to stations located in proximity to the monitoring area.

Correlation analyses were performed to explore potential relationships between the two climatic conditions and the dynamics of the host–parasitoid system. Specifically, the association between APR% and the average percentage of OSW per leaf was examined in relation to the average temperatures (°C) and cumulative rainfall (mm) recorded over the sampling periods. Analyses were conducted using MATLAB R2024b (version 9.17) [28]. Two statistical approaches were employed:

- Pearson’s correlation coefficients were calculated to assess the relationships between APR% and the climate variables.
- Simple linear regression models were fitted using the *‘fitlm’* function to explore and visualize potential trends.

The results were displayed as regression plots with 95% confidence bounds, and the statistical significance of the correlations was assessed based on *p*-values, with a significance level set at 0.05. Model selection was performed using Akaike’s Information Criterion (AIC) to assess both the goodness of fit and complexity of different linear regression models. The relative difference in AIC (∆AIC), defined as the difference between each model’s AIC and the lowest AIC observed, was used to determine the level of support for each model. Models with a ∆AIC less than 2 were considered to have substantial support and to be approximately equivalent in explaining the data [29]. A total of four simple linear regression models were applied, each incorporating a single climatic variable (either average temperature or cumulative rainfall) in relation to one biological variable (APR% or OSW population). Interaction terms between independent variables were not included, as the analysis was deliberately limited to univariate models to isolate the effect of each climatic factor.

## 3. Results

### 3.1. Parasitoid Collection and Characterization

**Figure 1:**
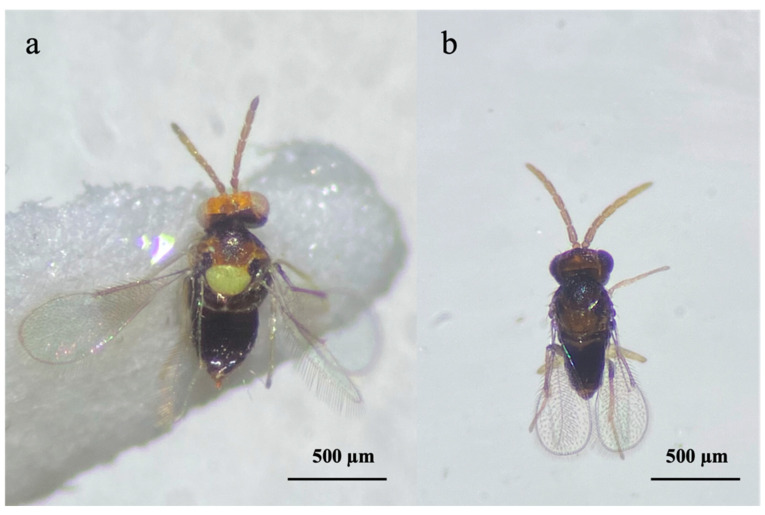
*Encarsia smithi*: (**a**) female; (**b**) male.

**Figure 2:**
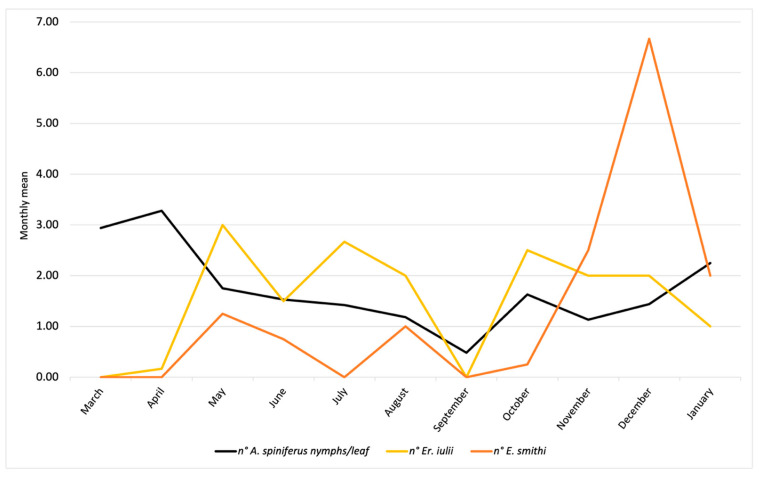
Population trends of *A. spiniferus* and its parasitoids (*Er. iulii* and *E. smithi*) observed during 2024 and early 2025 in Portici (NA).

Field observations revealed the presence of multiple parasitoid species associated with OSW populations. In addition to the predominant activity of *Er. iulii*, specimens of *E. smithi* were recovered from isolated fourth instar nymphs of *A. spiniferus* (Figure 1 and Figure 2).

**Figure 3:**
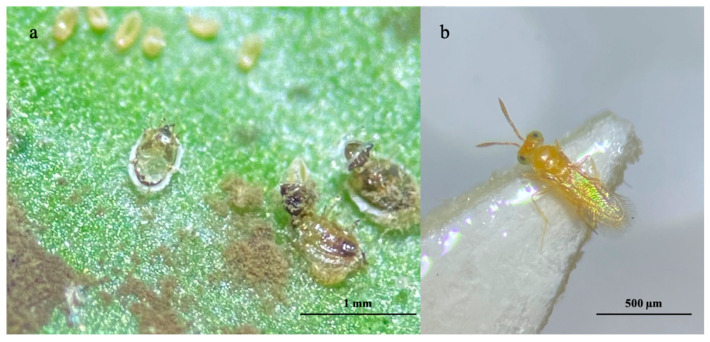
*Cales noacki*: (**a**) exit hole on OSW 3rd instar nymph; (**b**) adult female.

In addition, limited parasitoid activity of *C. noacki* on second and third-instar nymphs was observed in the material collected in Grottammare (AP) on 30 September 2024 (Figure 3).

Morphological examination allowed the identification of *Er. iulii* and *E. smithi* from samples collected at different sites (Figure 1). In addition, molecular analyses of *E. smithi* yielded identical COI sequences (734 bp) from all analyzed specimens. BLAST comparisons showed 100% identity with *E. smithi* haplotype O-J (haplogroup I; GenBank accession number: AB786726), while no matches were obtained from the BOLD database (Table 2).

### 3.2. Active Parasitization Rate

**Figure 4:**
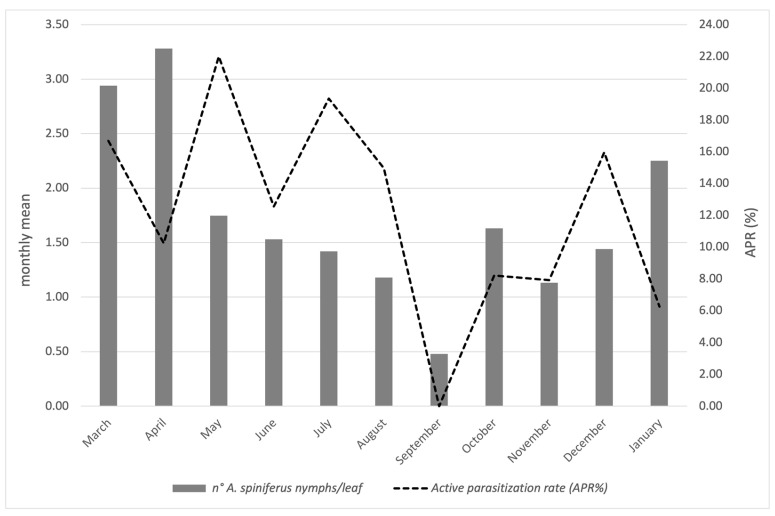
Infestation of *A. spiniferus*, along with the active parasitization rate attributable to *Er. iulii* during 2024 and early 2025 in Portici (NA).

The results referring to the province of Naples are reported in Figure 4 and concern the activity of *Er. iulii*, parasitoid is predominantly active at the investigated sites. The maximum APR% was 21.99%, recorded in May. A gradual reduction was observed from June (12.55%) until August. A sharp decline in both host abundance and parasitism was noted in September. Subsequently, the parasitization rate increased in October (8.21%), which was followed by a new peak (15.97%) in December, attributed to *Er. iulii*. Additionally, between May 17th and January 13th, 21 females and 16 males of *E. smithi* were obtained from the isolated material (Figure 2).

### 3.3. Phenology

The population trends of *A. spiniferus* are illustrated in Figure 2 and Figure 4. In Portici (NA), data show an increase in whitefly populations starting in March, reaching a peak in April, representing the highest level of infestation recorded in 2024. Subsequently, *A. spiniferus* densities decreased in May and June, in concomitance with the highest presence of *Er. iulii*. A second, but less intense, peak occurred in July/August, accompanied by a higher incidence of the parasitoid compared to April. In September, infestation levels dropped to a minimum, and no parasitoid activity was detected. During the following months, populations of both the whitefly and the parasitoid gradually increased, reaching new levels in January. Only in this final month has there been a reduction in *Er. iulii* activity was observed.

### 3.4. Impact of Climatic Conditions on Insect Population

Climate data are reported in Figures S1 and S2. The results of the linear regression analyses (Figures S3 and S4) did not reveal any statistically significant relationships. However, a moderate negative correlation was observed between the percentage of *A. spiniferus* per leaf and the average temperature (R = −0.3081; *p* = 0.064), suggesting an inverse proportional trend: as temperatures increase, the infestation level tends to decrease. Conversely, the correlation between OSW per leaf and rainfall was weakly positive (R = 0.1777; *p* = 0.293), indicating a slight tendency toward higher infestation rates under increased precipitation. In contrast, the correlations between the APR% and both average temperature (R = −0.0752; *p* = 0.654) and cumulative rainfall (R = −0.0475; *p* = 0.777) were weakly negative, suggesting no consistent linear relationship between these climatic variables and parasitization activity during the observed period. Model comparison using AIC and ∆AIC values showed that temperature-only models had the lowest AIC for both variables: 269.15 for APR% and 124.21 for OSW/leaf (∆AIC = 0 in both cases), indicating the strongest support among the tested climatic variables. For APR%, the rainfall-only model had an AIC of 269.28 (∆AIC = 0.13), and the combined model (temperature + rainfall) had an AIC of 270.94 (∆AIC = 1.79). For OSW/leaf, the rainfall-only model showed an AIC of 126.71 (∆AIC = 2.50), and the combined model had an AIC of 125.87 (∆AIC = 1.66). These results indicate that temperature was the most informative climatic predictor in both cases, although the relative differences among models were small. Despite this, none of the climatic variables showed statistically significant effects on APR% or OSW/leaf, suggesting a limited influence during the observed period.

## 4. Discussion

The present study reports for the first time the presence of *Encarsia smithi* in Europe, a species already considered a Beneficial Control Agent (BCA) in various biological control programs targeting *Aleurocanthus* spp. worldwide. Additional morphological, taxonomic, and distributional details are reported in Supplementary File S1. These findings, together with previous records of non-native antagonists of OSW in Italy, such as *Delphastus catalinae* and the possible non-native species *Eretmocerus iulii* [7,8,14], suggest that spontaneous introductions are underway, not deliberate by humans. For *Er. iulii*, the species was described for the first time in Italy, and currently, we cannot consider it with certainty to be allochthonous. In the current state of knowledge, and until reports from the same geographical areas of origin of the host are available, the activity of the aphelinid should be considered a new association.

Field observations revealed an increased biocenotic complexity of parasitoids associated with *A. spiniferus*. The population dynamics of the host and the two parasitoids (*Er. iulii and E. smithi*) (Figure 2 and Figure 4) indicate the currently limited presence of *E. smithi*, which is insufficient to support its establishment in the surveyed area. Nevertheless, the observed increase in *E. smithi* individuals during the late autumn–winter period may suggest a delayed activity phase compared to *Er. iulii*. The limited parasitization activity by *Cales noacki*, observed for the first time in association with OSW, represents a further potential contributor to the emerging complex of natural antagonists. Although *C. noacki* appears to have a rather broad host range [30], its association with OSW should be considered preliminary, pending further investigation. The new records require confirmation through long-term monitoring, and further studies are currently underway to verify the establishment of these parasitoids in the area.

Molecular analyses confirmed that *E. smithi* specimens collected in Italy belong to haplogroup I, the same group previously associated with OSW in Japan [11]. Given the absence of records of intentional releases and considering that commercial citrus imports generally involve defoliated fruit, it is plausible that *E. smithi* reached Italy through the movement of ornamental plants, possibly originating from already colonized regions such as Japan. Similar cases of unintentional introductions through the trade of vegetative material are day by day documented for other species, reinforcing the plausibility of this pathway also for beneficial insects [31,32]. An alternative hypothesis is that *E. smithi* arrived simultaneously with OSW but remained undetected during surveys conducted in previous years, possibly due to initially low population densities, a scenario that cannot be excluded. Notably, in Japan, haplogroup I has occasionally parasitized TSW [11]. Considering that TSW invaded Italy more recently and is still very limited in distribution [4,5], *E. smithi* may have entered via TSW infestations and subsequently shifted to OSW, its primary host. Thus, it is likely that the BCA (*E. smithi* in this case) was introduced from an already colonized, intermediate region such as Japan, rather than directly from its original range, a hypothesis already proposed in other similar contexts [33,34,35,36,37,38,39].

The results of the linear regression analysis showed no statistically significant correlation between average temperature or rainfall and either APR% or OSW infestation levels. Although a slight positive trend was observed between OSW per leaf and rainfall, this correlation was not statistically supported (*p* > 0.05) and should therefore be interpreted with caution. Nevertheless, periods of increased rainfall, particularly in October and December (Figure S3), appeared to coincide with both the vegetative regrowth phase of the host plants and a modest increase in OSW population levels. However, this is a qualitative observation not supported by quantitative plant growth measurements and thus cannot be used to infer a causal relationship. Regarding the interaction between OSW and its natural enemies, the activity of the two biological control agents (*Er. iulii* and *E. smithi*) appears to contribute to limiting OSW populations. Their effectiveness, however, may be partially influenced by abiotic conditions, such as elevated temperatures and reduced rainfall (Figures S1–S4), which could stress OSW without critically affecting parasitoid persistence. Model selection based on AIC and ΔAIC values suggested that temperature was the most informative climatic variable, although its explanatory power remained limited. Overall, these findings indicate that abiotic factors alone cannot fully explain the observed host-parasitoid dynamics, reinforcing the hypothesis that biotic interactions, particularly the activity of *Er. iulii* and *E. smithi*, are the primary drivers shaping OSW population trends during the study period.

The late-autumn and winter presence of *E. smithi* is particularly promising, although its establishment status remains to be assessed and will require multi-year monitoring across different sites to evaluate persistence, seasonal dynamics, and potential integration into the local parasitoid complex. In contrast, *Er. iulii* appears to be well acclimatized, with seasonal peaks in spring and early summer coinciding with marked reductions in *A. spiniferus* populations (Figure 2). The observed values are lower than those in previous periods of the year, both for the host and the aphelinid [8,14]. This decline is followed by a partial resurgence of the pest in late summer, aligned with renewed *Er. iulii* activity. The presence of eggs and ectophagous larvae on fourth-instar nymphs in early spring and late autumn (Figure S2) further suggests that *Er. iulii* may overwinter in these developmental stages. Furthermore, it is not implausible to hypothesize an interference between the ecto-endophagous *Er. iulii* and the strictly endophagous activity of *E. smithi*. The patterns observed in late summer suggest that the two parasitoids may occupy complementary ecological niches. This coexistence could enhance biocontrol efficacy and foster a stabilizing effect on the invaded ecosystem.

Invasive insect species increasingly threaten ecosystems worldwide, with climate change further facilitating the establishment and spread of thermophilic pests. Italy is particularly exposed due to its strategic position in international trade [40,41]. While the invasion and spread of phytophagous insects are well documented [42,43,44,45], the spontaneous response of indigenous and unintentionally introduced natural enemies remains less explored. Yet, such interactions often contribute to reducing invasive populations [33,34,46,47], though detailed analyses of these biocenoses are still scarce. The present case illustrates a now increasingly common scenario: a phytosanitary emergency that, over time, appears to evolve toward a new ecological equilibrium [34,36,48,49,50] with new arrivals of natural dispersal of antagonists and new associations between native and invasive species. What was once considered a threat may, through spontaneous biotic responses, shift into a system stabilized by naturally occurring antagonists. This situation evokes the dynamics of classical propagative biological control and raises the question of whether, under certain conditions, natural antagonisms can intervene before the intentional introduction of exotic BCAs becomes necessary.

## 5. Conclusions

This study documents the presence and potential progressive establishment of a diversified complex of natural enemies associated with *A. spiniferus* in Italy, primarily represented by the recently described *Er. iulii* and further enriched by the first European detection of *E. smithi*. The seasonal activity of *Er. iulii*, already widespread and apparently well-acclimated, appears to play a primary role in suppressing OSW populations. At the same time, the increasing presence of *E. smithi*, although right now still limited, may reflect a complementary dynamic that could strengthen natural control over time. The evidence presented here supports the emergence of a potentially functional and self-sustaining biological control system. The observed interactions among native and unintentionally introduced parasitoids suggest the development of an ecological equilibrium with relevant implications for long-term pest regulation. These findings also underscore the importance of unintentional biological control, a process often overlooked yet demonstrably capable of contributing to managing invasive species. Continued ecological monitoring will be essential to assess the persistence and synergistic potential of these interactions and to promote biologically driven solutions aligned with the principles of ecosystem resilience.

## Figures and Tables

**Table 1:** Localities, host plants, and frequencies of monitoring activities of *A. spiniferus* and its parasitoids.

Localities	Coordinates	Host Plants	Frequencies
Portici (NA)	40°48′55.4″ N 14°21′03.8″ E	*Citrus limon* *Citrus sinensis* *Citrus clementina*	Weekly (from March 2024 to January 2025)
	40°48′38.0″ N 14°20′31.0″ E		
Grottammare (AP)	42°59′19.6″ N 13°52′09.4″ E	*Citrus* × *aurantium* *Citrus sinensis*	Occasional, 30 September 2024

**Table 2:** *Encarsia smithi* specimens used for molecular identification through COI sequencing.

Specimen Code	Locality	Coordinates	Preliminary Identification	Accession Number
AA759	Portici (NA)	40°48′55.4″ N 14°21′03.8″ E	*E. smithi*	PV292129
AA760				PV292130
AA761				PV292131
AA762				PV292132
AA763				PV292133

## Data Availability

The datasets analyzed for this study are available in the public GitHub repository En_smithi at the following link: https://github.com/FrankNug/En_smithi.

## Supplementary Materials

The following supporting information can be downloaded at: https://www.mdpi.com/article/10.3390/insects16090891/s1, File S1. Detailed taxonomic and distributional information on *Encarsia smithi* [20,51,52,53,54,55,56,57,58,59,60,61,62,63,64,65,66,67,68,69,70,71,72,73]. Figure S1. Temperature trend in Portici during 2024–2025 surveys; Figure S2. Precipitation data in Portici during 2024–2025 surveys; Figure S3. Linear regression plots showing the correlation of OSW per leaf (%) with mean temperature (°C) (left) and rainfall (mm) (right); Figure S4. Linear regression plots showing the correlation of APR% with mean temperature (°C) (left) and rainfall (mm) (right).
