# Supplementary material for: First Detection of Encarsia smithi in Italy and Co-Occurrence with Eretmocerus iulii: A Case of Unintentional Introductions and New Associations with the Invasive Species Aleurocanthus spiniferus
Source: Insects. 2025 Aug 27;16(9):891. doi: 10.3390/insects16090891 (PMC12470831; doi:10.3390/insects16090891)
Supplement: Supplementary file 1 [file insects-16-00891-s001.zip › insects-3783387-supplementary.pdf]

Supplementary files

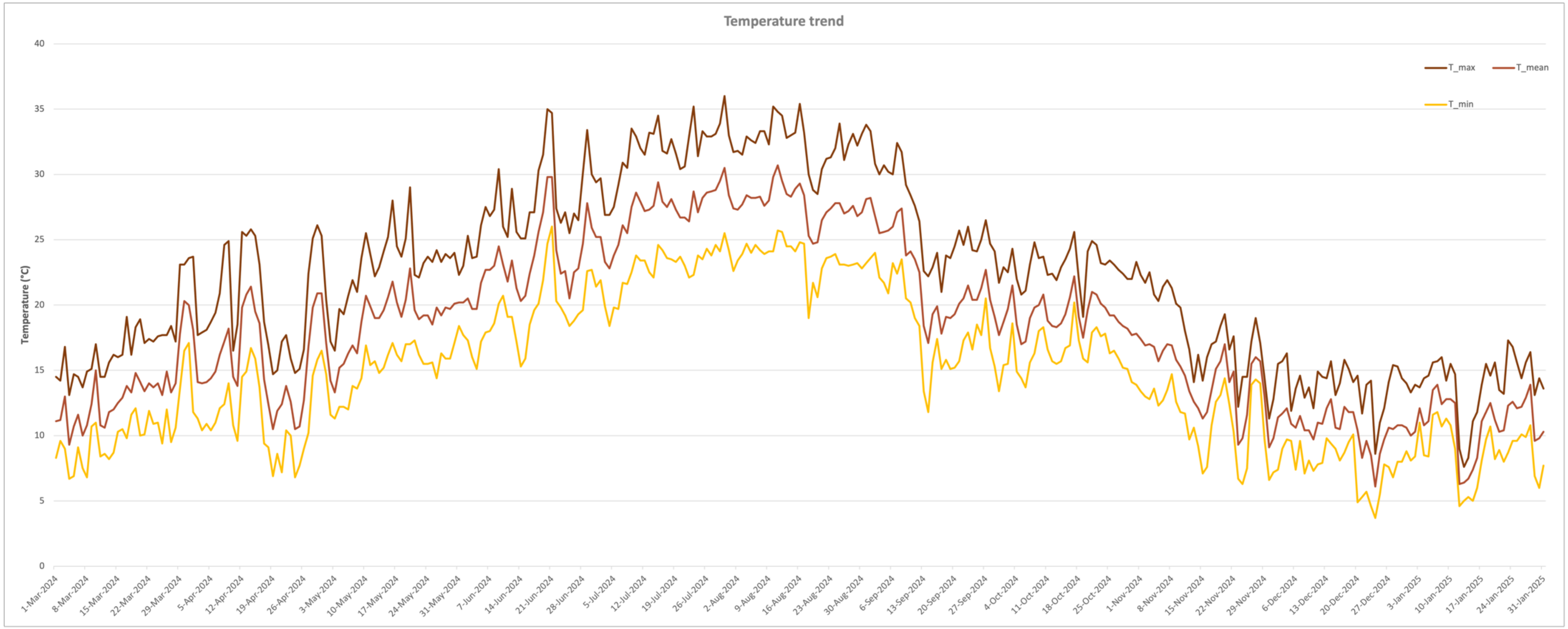

Figure S1. Temperature trend in Portici during 2024-2025 surveys

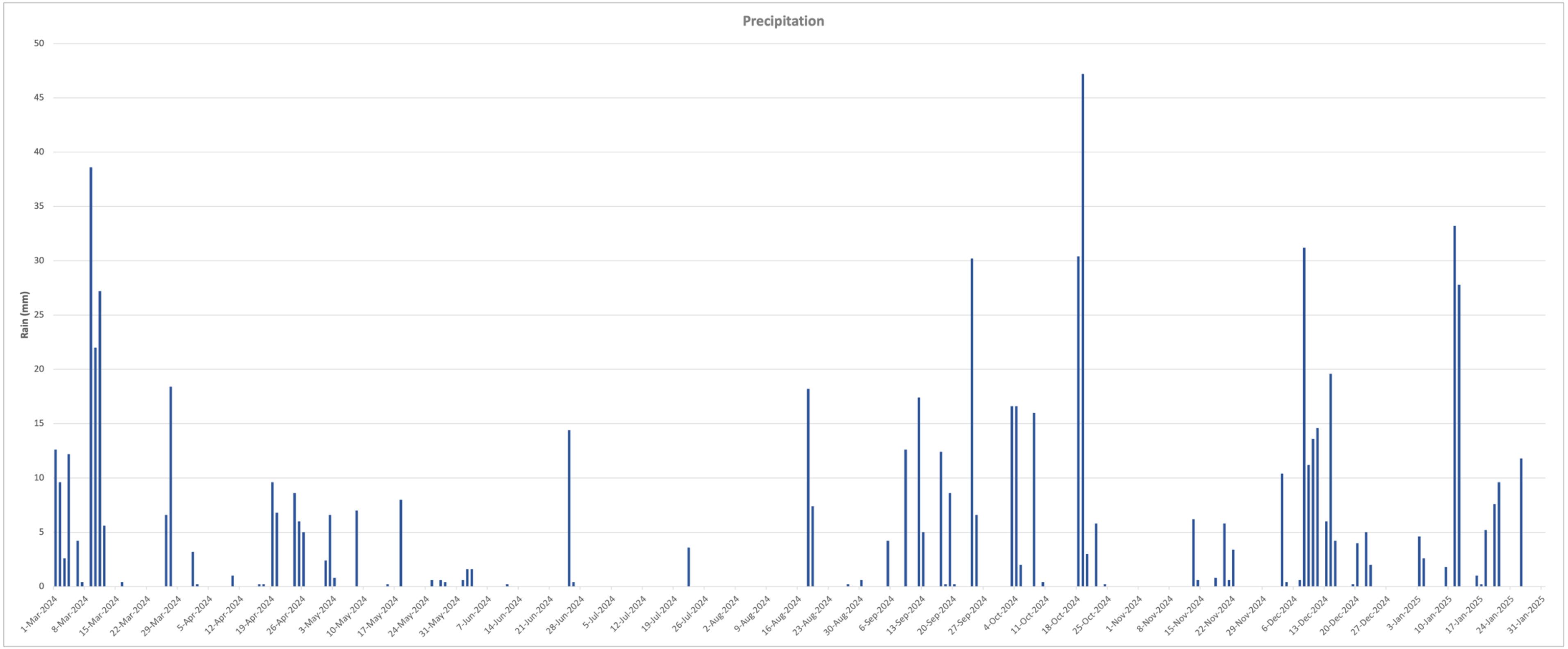

Figure S2. Precipitation data in Portici during 2024-2025 surveys

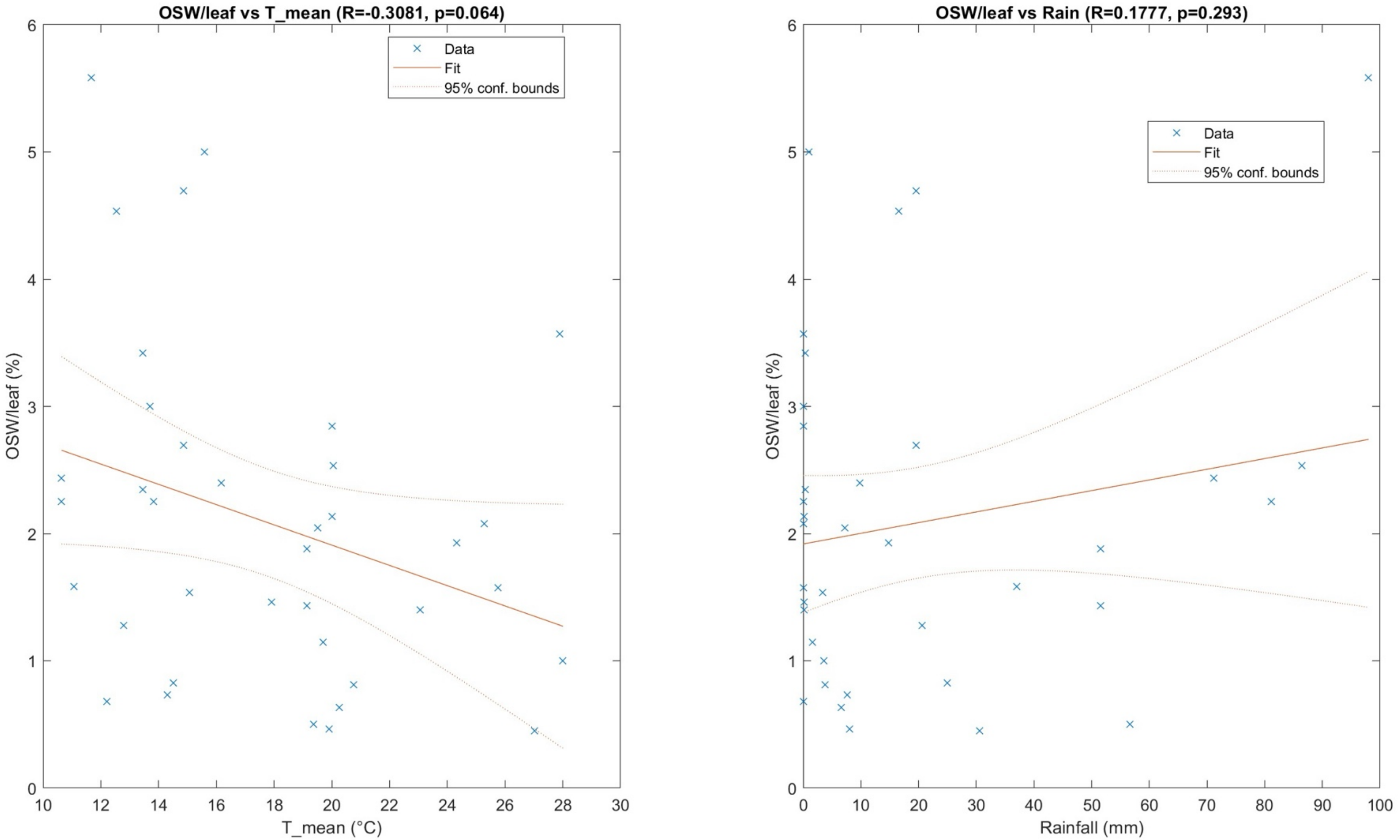

Figure S3. Linear regression plots showing the correlation of OSW per leaf (%) with mean temperature (°C) (left) and rainfall (mm) (right).

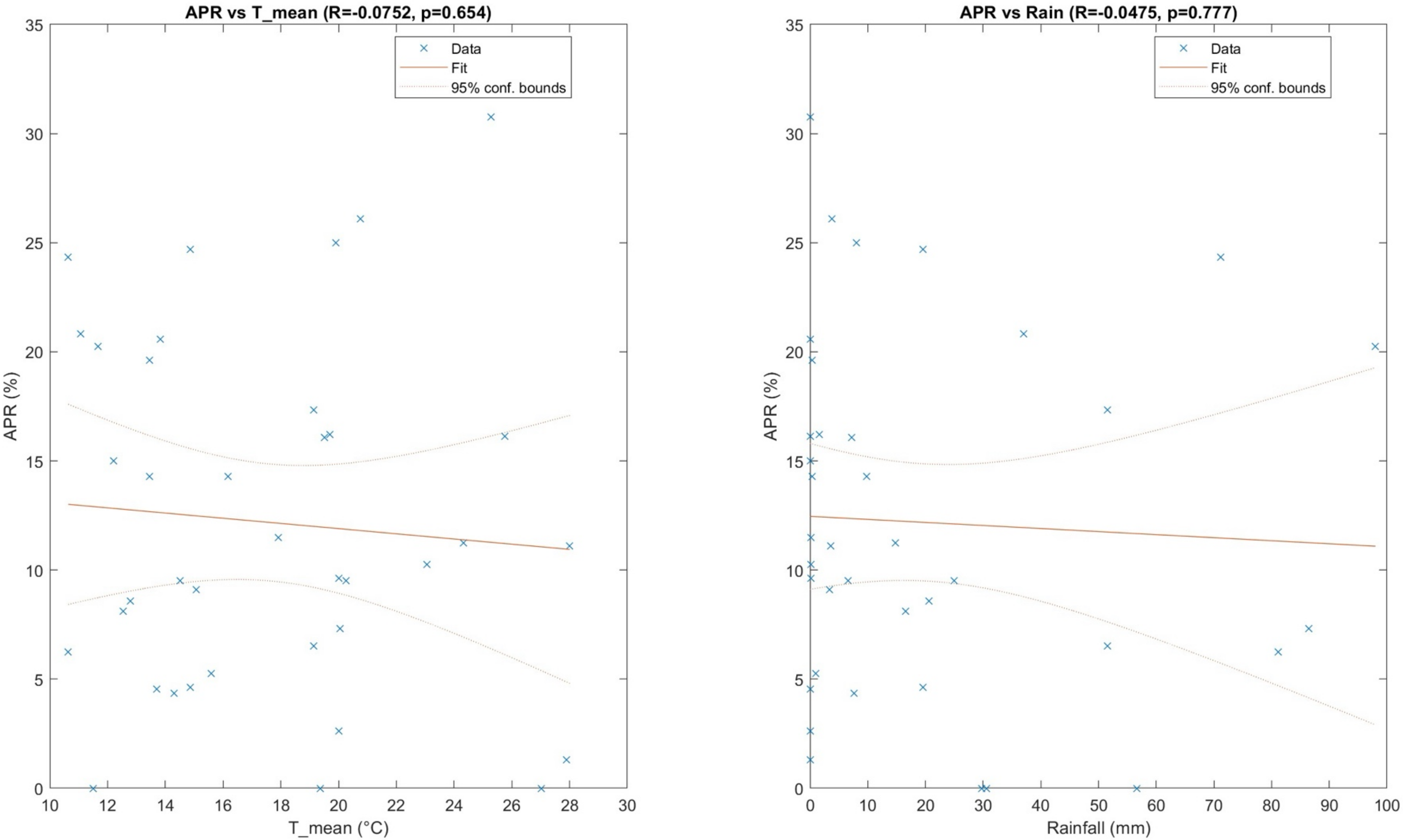

Figure S4. Linear regression plots showing the correlation of APR (%) with mean temperature (°C) (left) and rainfall (mm) (right).
