# Supplementary material for: First Detection of Encarsia smithi in Italy and Co-Occurrence with Eretmocerus iulii: A Case of Unintentional Introductions and New Associations with the Invasive Species Aleurocanthus spiniferus
Source: Insects. 2025 Aug 27;16(9):891. doi: 10.3390/insects16090891 (PMC12470831; doi:10.3390/insects16090891)
Supplement: Supplementary file 1 [file insects-16-00891-s001.zip › Supplementary_file S1.pdf]

File S1 Detailed taxonomic and distributional information on *Encarsia smithi*.

Encarsia smithi (Silvestri)

Original description: Silvestri [51] described the species based on ♀♀ and ♂♂ reared from *A. spiniferus* collected on citrus plants in different localities of China, including Canton, Macao, Fuzhou and Changsha. Additionally, he obtained some specimens also from *Aleurocanthus woglumi* Ashby collected in Colombo (Sri Lanka).

Distribution: Asia: Bangladesh, China, India, Japan, Maldives, Pakistan, Sri Lanka, Taiwan; North America/Caribbean: Cuba, Mexico, U.S.A.; Africa: South Africa, Swaziland (Eswatini); Oceania: Micronesia (UCD, 2023); Europe: Italy (this study).

Hosts: The known hosts of *En. smithi* are *Aleurocanthus citriperdus* Quaintance & Baker [52], *Aleurocanthus husaini* (Corbe) [52], *A. spiniferus* [53-64], *A. woglumi* [52,55,57,58,63,66-71], *Bemisia tabaci* (Gennadius) [57,58,72]

Diagnosis: This species was originally described by Silvestri under the name of *Prospaltella smithi*. Viggiani and Mazzone [73] proposed *Encarsia smithi* as a new combination and placed the species in the *smithi* group together with *Encarsia divergens* Silvestri 1926, *Encarsia ishii* (Silvestri), *Encarsia clypealis* (Silvestri), and *Encarsia merceti* Silvestri. The group is characterized by the morphology of the female antenna, which is not distinctly clavate, and by the first two funicular segments shorter than the subsequent ones [73]. According to Huang and Polaszek [20] and Myartseva and Evans [52], the female of *E. smithi* presents a head brown-yellow (Fig. 1a), the mesosoma yellow except for the pronotum, the mid lobe of the mesoscutum mostly, the anterior part of a side lobe of mesoscutum, axillae, mesopleuron, propodeum brown to dark brown. Midlobe of mesoscutum with 5 pairs of setae and with reticulate sculpture (Fig. 1b). Sidelobe of mesoscutum with 2 setae. The scutellar placoid sensilla are widely spaced, separated by a distance of about 4- 5 times the width of one sensillum. Gaster brown to dark brown, except the apex of 7_{th} tergite yellow. Third valvulae yellow. Antenna brown-yellow, 8-segmented and not distinctly clavate (Fig. 1c). Pedicel longer than first funicular segment (F1); F1 subquadrate, distinctly shorter than second and third funicular segments. Legs pale yellow except middle and hind coxae, hind femora brown to dark brown. Tarsal formula 5-5-5. Ovipositor shorter than the middle tibia and basitarsus combined (Fig. 1d). Forewing uniformly setose and infuscate below marginal vein, about 2.4-2.6x as long as wide (Fig. 1e); longest marginal fringe 0.23x wing width; submarginal vein with 2 setae; marginal vein with 6-7 setae along anterior margin; basal cell with 2 setae. Male coloration is like that of females, but with a body that is brown to dark brown, except for the yellow apex of the 7th tergite. Male antenna 8-segmented, with 6-segmented flagellum.
